# Cross-Talk between Probiotic Nissle 1917 and Human Colonic Epithelium Affects the Metabolite Composition and Demonstrates Host Antibacterial Effect

**DOI:** 10.3390/metabo11120841

**Published:** 2021-12-05

**Authors:** Karol Dokladny, John K. Crane, Alex J. Kassicieh, James B. Kaper, Olga Kovbasnjuk

**Affiliations:** 1 Department of Internal Medicine, Division of Gastroenterology and Hepatology, University of New Mexico Health Sciences Center, Albuquerque, NM 87106, USA; KDokladny@salud.unm.edu; 2 Department of Medicine, Division of Infectious Diseases, University at Buffalo, Buffalo, NY 14206, USA; jcrane@buffalo.edu; 3 University of New Mexico School of Medicine, Albuquerque, NM 87106, USA; ajkassicieh@salud.unm.edu; 4 Department of Microbiology and Immunology, University of Maryland School of Medicine, Baltimore, MD 21201, USA; jkaper@som.umaryland.edu; 5 Department of Medicine, Division of Gastroenterology and Hepatology, Johns Hopkins University School of Medicine, Baltimore, MD 21205, USA

**Keywords:** human colonoid monolayers, metabolome, glutamine, GABA, spermine, host anti-bacterial effect

## Abstract

Colonic epithelium–commensal interactions play a very important role in human health and disease development. Colonic mucus serves as an ecologic niche for a myriad of commensals and provides a physical barrier between the epithelium and luminal content, suggesting that communication between the host and microbes occurs mainly by soluble factors. However, the composition of epithelia-derived metabolites and how the commensal flora influences them is less characterized. Here, we used mucus-producing human adult stem cell-derived colonoid monolayers exposed apically to probiotic *E. coli* strain Nissle 1917 to characterize the host–microbial communication via small molecules. We measured the metabolites in the media from host and bacterial monocultures and from bacteria-colonoid co-cultures. We found that colonoids secrete amino acids, organic acids, nucleosides, and polyamines, apically and basolaterally. The metabolites from host-bacteria co-cultures markedly differ from those of host cells grown alone or bacteria grown alone. Nissle 1917 affects the composition of apical and basolateral metabolites. Importantly, spermine, secreted apically by colonoids, shows antibacterial properties, and inhibits the growth of several bacterial strains. Our data demonstrate the existence of a cross-talk between luminal bacteria and human intestinal epithelium via metabolites, which might affect the numbers of physiologic processes including the composition of commensal flora via bactericidal effects.

## 1. Introduction

Growing evidence demonstrates the importance of the interactions between intestinal microbiota and human colonic epithelium in health and pathophysiology. Over the last decades, we learned that gut microflora acting as probiotics, prebiotics, and via secreted metabolites modulates the intestinal barrier function, resulting in inflammatory diseases, obesity, hypertension, cardiovascular diseases, and colorectal cancer [1,2,3,4,5,6]. It influences neurologic and neurodegenerative disorders via the gut–brain axis [7,8]. The richness of microbiota itself is very much shaped on a daily basis and chronically by diet, lifestyle, microbial (fecal transplantation), and anti-microbial (antibiotics, etc.) therapies, etc. Numerous studies have addressed the important role of diet-dependent and diet-independent bacterial metabolites on the healthy metabolic status of the host intestinal and systemic physiology (kidney, cardiovascular, etc.), including short-chain fatty acids, secondary bile acids, indole, etc. Overall, modulation of these multiple host physiologic and disease-related pathways by intestinal microorganisms occurs mainly via the exchange of small molecules between the intestinal lumen and human epithelium [4,9,10].

However, luminal metabolites derived from human epithelium and the microbial influence on their composition are less studied [11]. This is partially due to technical difficulties in the separation of human-derived vs. microbe-derived entities. Additionally, host-specific genetic differences, gene regulation, as well as individual differences in microbiota (persistent or temporary, driven by diet and other factors) may substantially add to the complexity of outcomes. Adult stem cell-derived human colonoid monolayer (HCM) cultures [12,13,14,15] are a suitable model to characterize the host-derived metabolites, address the role of commensal bacteria in shaping the luminal metabolome, and identify the source of each metabolite, host- or bacteria-derived.

Here, using HCM alone or in co-culture with well-characterized probiotic *E. coli* bacteria Nissle 1917, we analyzed the composition of small molecules secreted by human intestinal epithelium into the apical and basolateral compartments. We also characterized the effect of host-produced metabolites on the growth of intestinal luminal bacteria, including Nissle 1917, Enterohemorrhagic *Escherichia coli* (EHEC), and several other bacterial strains.

## 2. Results

### 2.1. Interaction between Human Colonoids and Nissle 1917 Affects the Amino Acid Composition in Apical and Basolateral Conditioned Media

To test the composition of small molecules in HCM and Nissle 1917 co-culture, HCM incubated in differentiation media were apically infected with low initial inoculum (10^3^ CFU) of the Nissle 1917 bacteria for 48 h. The apical conditioned media (ApCM) and basolateral conditioned media (BlCM) from the infected monolayers were collected for metabolome analysis. Conditioned for 48 h, ApCM and BlCM from uninfected HCM served as the control. To characterize the metabolome of Nissle alone, the media from 48 h bacterial culture (10^3^ CFU) grown in colonoid differentiation media was also subjected to metabolome analysis. Uninfected colonoid differentiation (Diff) media was used as the control. In total, six types of media samples were subjected to small molecule analysis (Supplementary Materials Table S1).

**Figure 1:**
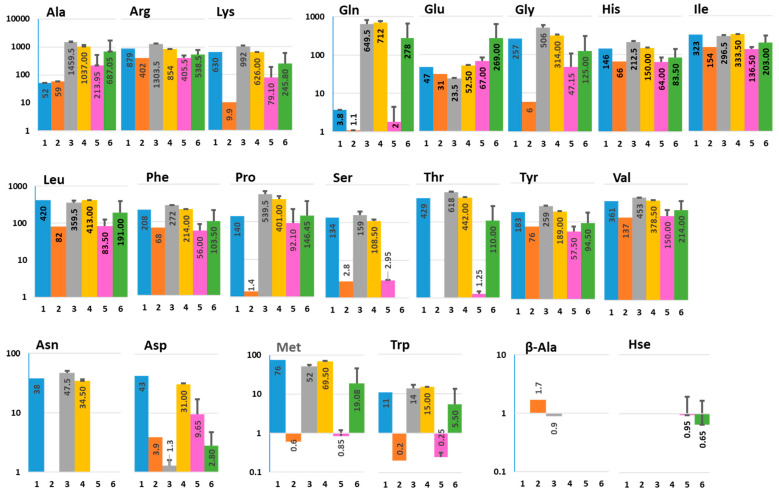
Effect of Nissle 1917 infection on the amino acid composition in ApCM and BlCM collected from differentiated HCM. The composition and concentration of amino acids were measured in media collected from six different conditions: (1) Diff media alone (control); (2) Nissle 1917 cultured for 48 h in Diff media (control); (3) ApCM from uninfected differentiated HCM; (4) BlCM from uninfected differentiated HCM; (5) ApCM from differentiated HCM infected for 48 h with Nissle 1917; (6) BlCM from differentiated HCM infected for 48 h with Nissle 1917. X axis—experimental conditions; Y axis—concentration (µM; logarithmic scale). The number on each bar corresponds to average metabolite concentration (µM) obtained from two independent technical replicates; Data presented as AVG ± SD.

We detected 21 amino acids (AAs) that could be classified based on changes of their concentration by host alone, bacteria alone, or by bacteria-infected host (Figure 1 and Table S1). The majority of detected AAs were present in Diff media, except β-alanine and homoserine. The concentration of many AAs, except Glu and Ala, is substantially lower in the presence of Nissle compared to Diff media, indicating that they could be consumed by bacteria. Additionally, many AAs are secreted apically and basolaterally by differentiated HCM, including Gln, Ala, Arg, Gly, His, Lys, and Pro.

Glutamine (Gln), a conditionally essential amino acid, is considered the most important fuel for intestinal tissue [16] and it is always present in mammalian cell culture media to support cell growth. Surprisingly, the concentration of Gln in colonoid apical conditioned media (ApCM; condition # 3) and basolateral conditioned media (BlCM; condition #4) was much higher (~160 fold) than in Diff media alone (condition #1), indicating that differentiated colonoids secrete Gln apically and basolaterally. In contrast to ApCM from uninfected HCM (condition #3), the Gln concentration in ApCM from the Nissle-infected colonoids (condition #5) was substantially lower (~320 fold). Nissle infection did not affect Gln in BlCM (condition #6) compared to this in BlCM from uninfected HCM (condition #4). Nissle grown in Diff media (condition #2) did not display substantial differences in the Gln concentration when compared to Diff media alone (condition #1), indicating that these bacteria might inhibit apical glutamine secretion from colonoids, rather than consume the apical glutamine.

**Figure 2:**
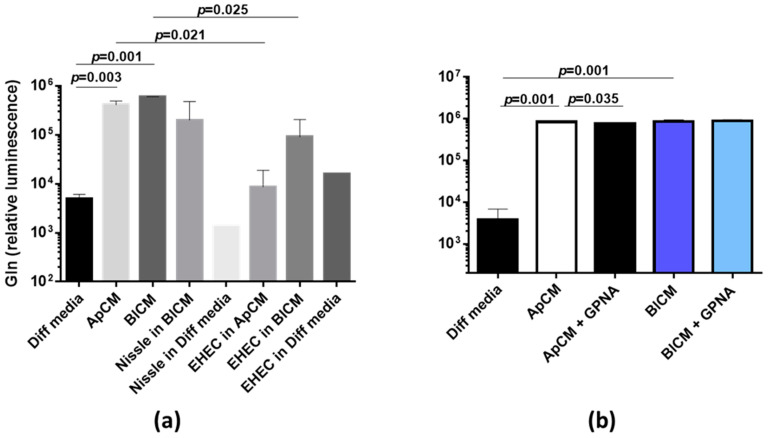
Luminal bacteria modulate the Gln secretion from HCM. (**a**) Gln is secreted into apical conditioned media (ApCM) and basolateral comdtioned media (BlCM) by differentiated uninfected HCM, and Gln secretion from HCM is inhibited by pathogenic Enterohemorrhagic *E. coli* (EHEC) O26:H11 bacteria added apically (10^3^ CFU) for 2 days. Uninfected Diff. medium was used as the control. Data presented as mean ± SEM, *n* ≤ 3 per each condition (except data on Nissle and EHEC grown in Diff. media; *n* = 2) (**b**) Treatment of confluent differentiated uninfected HCM for 3 days with 100 µM of L-γ-Glutamyl-p-nitroanilide(GPNA), a specific SLC1A5 inhibitor in apical and basolateral compartments significantly decreases Gln apical but not basolateral concentration. Gln was measured in all collected media using ELISA bioluminescent assay; Data presented as mean ± SEM; *n* = 3 per condition.

We confirmed these metabolome data using the Gln ELISA assay and found that ApCM and BlCM collected from differentiated HCM have a significantly higher Gln concentration compared to the Diff media alone (Figure 2a and Figure S1). In contrast, the Gln concentration in conditioned media (CM) from undifferentiated HCM was significantly lower than in undifferentiation (growth) media alone (Figure S1), indicating that proliferating colonoids consumed the Gln from the media, as was expected due to previous publications [16]. Nissle infection did not decrease Gln in BlCM (Figure 2a), similarly to what we detected in the metabolite screen (Figure 1). Nissle are probiotic bacteria [17,18]. We tested whether intestinal pathogens would similarly affect the Gln. Infection of human colonoids with *Enterohemorrhagic E. coli* (EHEC) O26:H11 strain [19] significantly deceased Gln in ApCM (Figure 2a). In contrast to Nissle, EHEC significantly decreased the basolateral Gln concentration. These data further suggest that Gln is secreted from differentiated human colonocytes and both types of bacteria interfere with its secretion.

Gln is the most abundant amino acid in plasma and has numerous roles in humans and other mammals. To fulfill these roles, Gln has to be taken into or released from cells by membrane transporters. Currently, at least 14 amino acid transporters have been identified to transport Gln, including SLC1, 6, 7, and 38 family members. However, most of these transporters mediate the Gln influx. SLC1A5 (ASCT2), a sodium-dependent amino-acid antiporter, has been implicated in the efflux of Gln [20]. The transport reaction is sodium-dependent antiport in which Gln is co-transported with Na+ in exchange with another neutral amino acid. SLC1A5 is highly expressed at the brush border of human intestinal epithelial cells [21]. Thus, we reasoned it might be a good candidate for Gln secretion from differentiated HCM. Indeed, treatment of HCM with SLC1A5 inhibitor GPNA significantly decreased the Gln apical concentration, indicating SLC1A5 contribution to apical Gln secretion (Figure 2b). However, inhibition was moderate, suggesting additional mechanisms of apical Gln efflux. GPNA did not affect the basolateral Gln concentration, indicating an SLC1A5-independent mechanism of its basolateral secretion.

Similarly to Gln, the non-essential amino acid Ala was substantially elevated in ApCM and BlCM from uninfected (~20 fold; conditions #3 and 4) compared to this in uninfected Diff media (condition #1). The Ala was downregulated in ApCM (~6 fold; condition #5) from Nissle-infected HCM. Again, Nissle did not metabolize Ala from Diff media, indicating that bacteria may affect its apical secretion.

Another group of non-essential AAs, including Asn, Asp, and Ser and several essential AAs (His, Ile, Val, Lue, Met, Thr, Trp, and Tyr), were metabolized by bacteria, which might contribute to their substantially lower concentrations in ApCM from infected HCM in comparison to ApCM from uninfected cultures (Figure 1). These AAs were also downregulated in BlCM from Nissle-infected HCM. This is particularly well illustrated by Asn and Ser, which were below detection levels in BlCM from infected HCM. In contrast, Glu was substantially elevated in BlCM from Nissle-infected colonoids. These data suggest the complex communication between apically present commensals and colonic epithelium, which may use small molecules to transduce the luminal signals to the numerous cell types present in the intestinal lamina propria.

In contrast to AAs described above, β-alanine was absent from Diff media but was detected in the presence of Nissle, and in ApCM and BlCM from uninfected HCM. Surprisingly, it was below detection in host-commensal co-cultures. On the contrary, homoserine was detected only in the infected HCM, both apically and basolaterally.

### 2.2. Nissle 1917-Produced Metabolites

Several metabolites (Table S2), mainly related to energy production (3-Phosphoglyceric acid, Acetyl CoA-divalent, Glycerol 3-phosphate, NAD+) and RNA synthesis (AMP (adenosine monophosphate), UMP (Uridine monophosphate), and CMP (cytidine monophosphate)) were detected exclusively in Nissle-grown Diff media but appeared undetectable in the Nissle-infected colonoids. It is well established that numerous microorganisms, including bacteria, produce these small molecules, which are essential for growth and division [22,23]. For example, G3P can facilitate microorganism growth by providing a combined source of phosphorus, carbon, and energy. Acetyl-CoA production by bacteria appeared very early in evolution and plays an important role in cellular respiration and the tricarboxylic acid (TCA) cycle. 3-Phosphoglyceric acid (3-PG) exists in all living species, ranging from bacteria to humans, and is the conjugate acid of glycerate 3-phosphate, a precursor for serine, which, in turn, can create cysteine and glycine. Nucleotides AMP, UMP, and CMP are the components in the synthesis of RNA. Additionally, AMP plays an important role in many cellular metabolic processes, being interconverted to ADP and/or ATP. It has to be determined why these metabolites were undetectable in the media from HCM (infected or uninfected).

### 2.3. Nucleosides and Their Precursors

**Figure 3:**
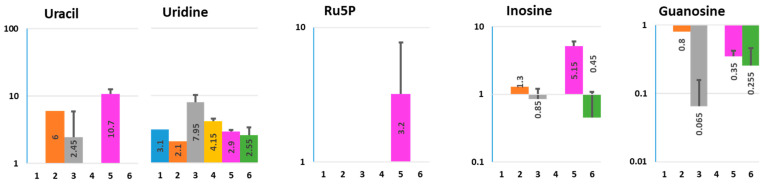
Effect of Nissle 1917 infection on the composition of nucleosides and their precursors in ApCM and BlCM collected from differentiated HCM. Ru5P—Ribulose-5-phosphate; X axis—experimental conditions, as described in Figure 1 and Table S1; Y axis—concentration (µM, logarithmic scale). Data presented as AVG ± SD.

Nucleosides inosine, guanosine, uridine, and its precursor uracil were detected in Nissle-grown Diff media (Figure 3 and Table S1). Diff media alone was most likely the main source of uridine, and this would explain its presence in both apical and basolateral media from uninfected and Nissle-infected HCM. However, uridine precursor uracil was produced by bacteria and secreted apically from uninfected colonoids, thus being present in apical media from Nissle-infected monolayers.

Guanosine and inosine, in addition to being produced by bacteria and apically secreted by colonoids, were also detected in BlCM from Nissle-infected cultures, indicating possible differences in their handling by intestinal epithelial cells in the presence of the commensals. The inosine concentration was substantially higher in ApCM from Nissle-infected HCM, than in media from either bacteria or colonoids alone. Inosine is one of the rare nucleosides found in nucleic acids. Inosine and guanosine nucleosides are sources of the umami flavor, with attributed beneficial health effects that have renewed commercial interest in nucleoside fermentations [24]. It has been shown that some dietary supplementation resulted in the enrichment of microbiota-derived inosine or exogenous inosine treatment could activate PPARγ signaling in human colonic epithelial cells and protect against DSS-induced colitis through the improvement of adenosine 2A receptor (A_2A_R)/PPARγ-dependent mucosal barrier functions. Thus, the interactions between the gut microbiota, inosine, and A_2A_R/PPARγ play an important role in the maintenance of intestinal homeostasis. This may represent a novel approach for colitis prevention via manipulation of the gut microbial purine metabolite [25,26].

Ribulose-5-phosphate, the product of the aerobic segment of pentose phosphate pathway that is usually converted to ribose-5-phosphate necessary for synthesis of nucleotides and nucleic acids, was detected only in ApCM from Nissle-colonoid co-culture and the source of it (bacterial, or host, or both in the co-culture) has to be determined.

### 2.4. Organic Acids

#### 2.4.1. 3-Hydroxybutyric Acid (3-HBA)

**Figure 4:**
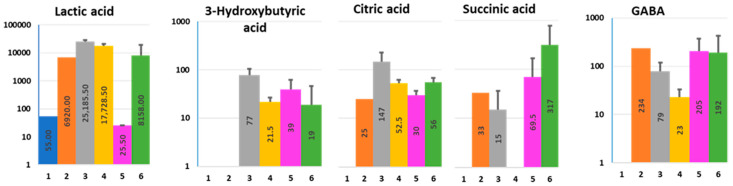
Effect of Nissle 1917 infection on the composition of organic acids in ApCM and BlCM collected from differentiated HCM. X axis—experimental conditions, as described in Figure 1 and Table S1; Y axis—concentration (µM, logarithmic scale). Data presented as AVG ± SD.

3-HBA is secreted apically and basolaterally, but its apical concentration in Nissle-colonoid co-culture appears substantially lower compared to this in colonoids alone (Figure 4 and Table S1).

In mammalian cells, 3-HBA is formed as a product of fatty acid oxidation and can therefore be used as an energy source in the absence of glucose [27]. 3-HBA is an important metabolite and a signaling molecule that can influence gene expression, lipid metabolism, neuronal function, and the overall metabolic rate. Some of these effects are the direct 3-HBA effects, while others are indirect effects, regulated by the metabolites into which 3-HBA is converted. One of the most important regulatory functions of 3-HBA is the inhibition of the activity of histone deacetylases and thus the epigenetic regulation of many genes. In microorganisms, 3-HBA mainly serves as a substrate for the synthesis of polyhydroxybutyrate, which is a reserve material [27]. It has been shown that wild-type bacteria, including *E. coli*, normally do not produce extracellular 3-HBA [28]. Indeed, Nissle alone did not produce the detectable amount of 3-HBA (Figure 4). This suggests that similarly to other microorganisms [27], Nissle might utilize 3-HBA as the reserve source of carbon and energy, thus lowering its concentration in ApCM. However, the mechanisms of 3-HBA secretion by intestinal epithelium as well as its utilization by Nissle, including the transporter responsible for the uptake of extracellular 3-HBA by bacteria, require further investigation.

#### 2.4.2. Lactic, Citric, and Succinic Acid

Although citric acid and lactic acid were both secreted by bacteria alone and to a greater extent by colonoids alone (apically and basolaterally), Nissle infection substantially inhibited their apical concentration (Figure 4). In contrast, bacteria increased the apical and basolateral concentration of succinic acid.

Lactic and citric acids can synergistically inhibit the growth of pathogenic bacteria and spoilage organisms, thus representing a host defense mechanism. It has been shown that a mixture of lactic and citric acids significantly inhibits the growth of EHEC strain O157:H7 or *Salmonella Typhimurium*, demonstrating the potential for the inactivation of foodborne pathogens [29]. Our data indicate that bacteria might use some mechanism to reduce the apical concentration of both acids, thus evading the host defense and improving bacterial growth and survival. Bacterial _L_-lactate permease was found to mediate the uptake of lactate [30]. Lactate can be utilized by *E. coli* as a sole source of carbon and energy [31]. A recent excellent review discussed in detail the lactate transporters in human and bacterial cells, the multiple roles of host and bacteria-produced lactate in host-microbe, and the interactions that influence cancer, autoimmunity, and inflammation [32].

The succinic acid concentration substantially increased apically and basolaterally in Nissle-colonoid co-cultures compared to corresponding monocultures (Figure 4). Succinate is a metabolic intermediate of the TCA cycle within the host cells. An increase in mitochondrial succinate occurs mainly from the elevation of glutamate synthesis, which is ultimately converted to succinate as part of the Krebs cycle [33]. Elevation of succinate is in good agreement with our data showing the elevation of glutamine (Figure 1), the glutamate precursor. Succinate is also produced in large amounts during bacterial fermentation of dietary fiber [34]. A question remains whether bacteria or host cells mainly contribute to elevated apical and basolateral levels of succinate. The studies comparing germ-free mice with control mice have shown that fecal succinate levels are almost undetectable in the former, which points to gut microbiota as the predominant source of luminal succinate [34]. Elevated succinate levels within the gut lumen have been reported in association with dysbiosis, as well as in patients with inflammatory bowel disease (IBD) and animal models of intestinal inflammation. Succinate may act as a pro-inflammatory stimulus [33,35], and its basolateral elevation in the infected colonoids might contribute to the inflammatory responses by lamina propria cells. Thus, an increase in both serum and intestinal succinate levels has been reported in patients with Crohn’s disease when compared with healthy subjects [36]. The mechanism of succinate release into the basolateral media thus might be an important target in inflammatory diseases. Succinate was also described as a virulence factor that might exacerbate enteric infections [37]. Likewise, succinate-rich environments are sensed by EHEC pathogens to activate virulence factors [38]. Elevation of intracellular succinate levels also suggests a metabolic switch from oxidative phosphorylation to glycolysis. Future studies are needed to determine how the extracellular succinate is elevated in the presence of bacteria and what role the extracellular succinate plays in host–microbe interactions.

#### 2.4.3. Gamma-Aminobutyric Acid (GABA)

Nissle is the major contributor to the apical GABA pool, though the colonoids were also found to contribute here (Figure 4). These data confirm the published observation that microbiota (*E. coli, Bacteroides*, etc.) produce GABA from decarboxylation of glutamate, or from other precursors as arginine, putrescine, and secrete GABA for alkalinization [39,40]. Therefore, the well-described positive effects of glutamine on enterocytes and intestinal integrity might be partly attributed to the effects of its metabolite GABA.

GABA is a well-characterized neurotransmitter [40]. However, it might play a much broader role in host–commensal interaction. It has been shown that GABA selectively increases the expression of MUC1, a cell surface mucin that prevents the adhesion of microorganisms, due to its size and negative charge [41]. GABA has also been detected in the cytoplasm and brush border of epithelial cells in the rat jejunum and colon [42,43], which is in good agreement with our findings. Microbiota might also regulate GABA synthesis by the host [44,45]. The striking difference between Nissle-colonoid co-culture and colonoid monoculture is a substantial increase in GABA at the basolateral side of epithelium, indicating that probiotic bacteria might not just produce GABA, or stimulate its production from a host, but modulate its trans-mucosal transport, delivering more GABA to gut mesenteric and submucosal neuronal plexuses as well as systemically. Thus, GABA present in the gut lumen could play a major role in the gut–brain axis of systemic communication. Importantly, a recent human study found that transplant of fecal microbiome from lean to obese individuals resulted in increased levels of GABA in plasma [46], further illustrating how host–microbial interactions are transmitted systemically.

### 2.5. Toxins

**Figure 5:**
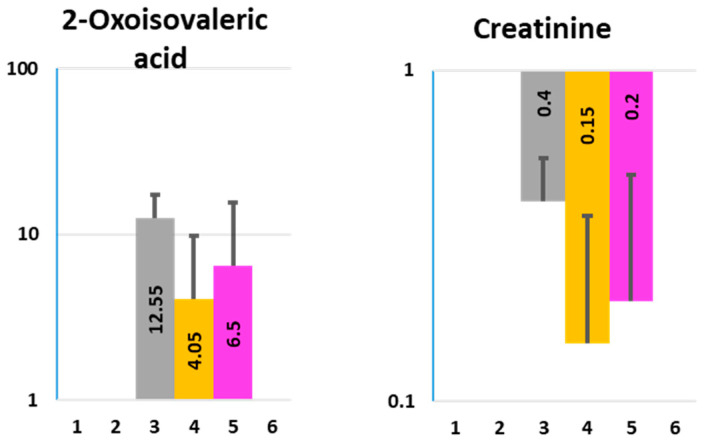
Effect of Nissle 1917 infection on the concentration of two toxins, 2-Oxoisovaleric acid and creatinine in ApCM and BlCM collected from differentiated HCM. X axis—experimental conditions, as described in Figure 1 and Table S1; Y axis—concentration (µM, logarithmic scale). Data presented as AVG ± SD.

2-Oxoisovaleric acid (also known as α-ketoisovaleric acid) and creatinine are both secreted by uninfected HCM apically and basolaterally, whereas Nissle infection substantially decreases their apical and basolateral concentrations to below detection levels (Figure 5 and Table S1).

α-Ketoisovaleric acid is a neurotoxin leading to severe brain damage [47,48]. Chronically high levels of α-ketoisovaleric acid are associated with maple syrup urine metabolic disorder caused by a buildup of the branched-chain amino acids (leucine, isoleucine, and valine) and their toxic by-products (ketoacids) in the blood and urine. This leads to a depletion of glutamate and a subsequent reduction in the concentration of brain glutamine, aspartate, alanine, and other amino acids. The result is a compromise of energy metabolism because of a diminished rate of protein synthesis [49]. A substantial decrease of basolateral 2-oxoisovaleric acid in the presence of Nissle might demonstrate a positive effect of this probiotic in preventing systemic toxicity. If confirmed in vivo, this could be another example of microbe-modulated protective gut–brain communication.

Creatinine, the waste product of the creatine catabolism in muscles or food-derived, is filtered mainly by the kidneys [50]. Elevated blood creatinine is thought to be the major cause of renal failure. It has been suggested that colonization of the gastrointestinal tract by creatinine-consuming bacteria that express creatininase might increase an extra renal clearance contributing to gut–kidney cross-talk [50]. Thus, Nissle, by lowering the creatinine in BlCM, may decrease the systemic burden of creatinine (Figure 5). However, the role of probiotics in reducing kidney toxicity by creatinine is controversial [51].

### 2.6. Polyamines

**Figure 6:**
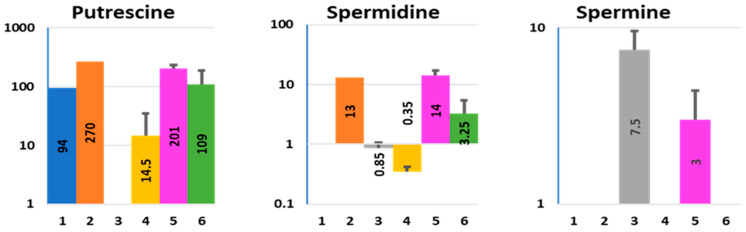
Effect of Nissle 1917 infection on the polyamine composition in ApCM and BlCM collected from differentiated HCM. X axis—experimental conditions, as described in Figure 1 and Table S1; Y axis—concentration (µM, logarithmic scale). Data presented as AVG ± SD.

Putrescine, spermidine, and spermine were found in different combinations in the tested media (Figure 6 and Table S1).

Putrescine was present in Diff media and was further elevated in the presence of bacteria. These data would explain its apical and basolateral presence in colonoid-Nissle co-cultures. However, it was below the detection level in ApCM from uninfected colonoids, suggesting that luminal putrescine can be consumed by host cells. In contrast, Nissle were the major source of spermidine detected in both apical and basolateral media from colonoid-bacterial co-cultures.

**Figure 7:**
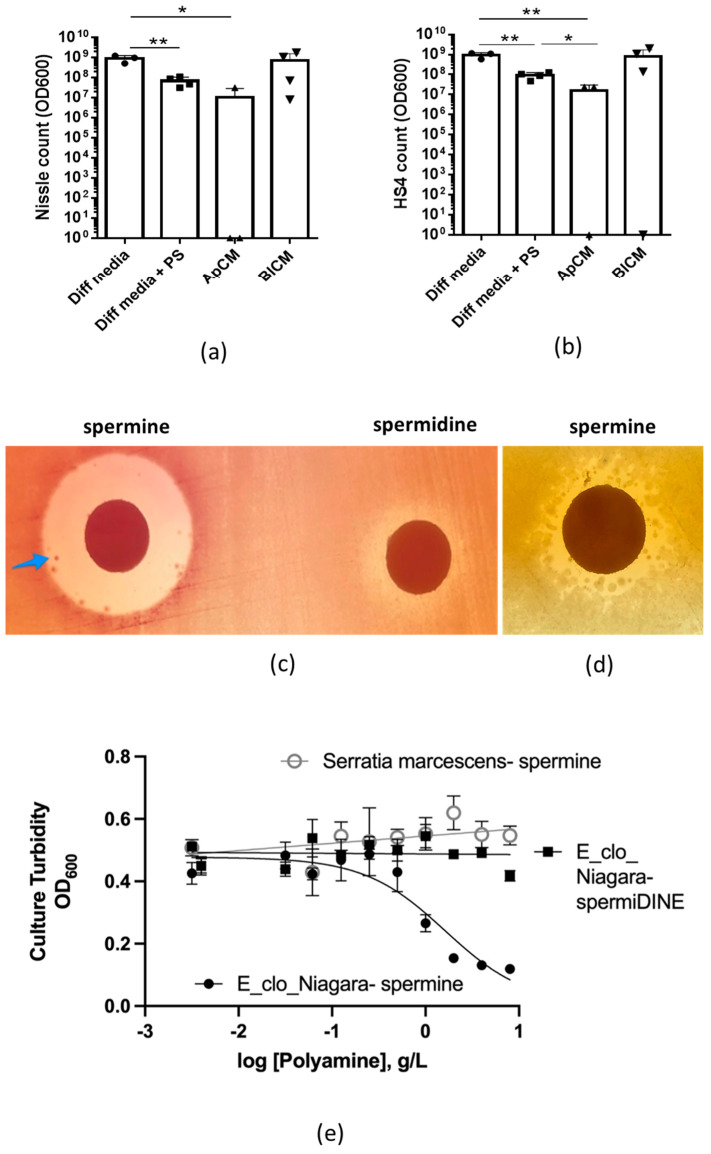
ApCM has broad antibacterial properties, which are attributed to the presence of spermine. ApCM significantly inhibits the growth of (**a**) Nissle 1917 strain *E. coli* HS4 strain and (**b**) *E. coli* HS4 strain compared to the bacterial growth in BlCM and in Diff media. For these experiments, 10^3^ CFU of either Nissle 1917 or HS4 were incubated for 48 h with either Diff media, or Diff media supplemented with 100U penicillin/streptomycin, or ApCM, or BlCM collected from differentiated HCM. The antibacterial effect of ApCM was similar to the effect of penicillin and streptomycin mixture present in Diff media; Data presented as mean ± SEM; *n* ≤ 3 per each tested conditions; *—*p* ≤ 0.05; **—*p* ≤ 0.01; (**c**) Exogenous spermine but not spermidine substantially inhibits the growth of laboratory *E. coli* JLM281 plated on MacConkey agar. Blue arrow shows partially spermine-resistant inlier colonies within the zone of inhibition; (**d**) Exogenous spermine inhibits the growth of *Enterobacter cloacae*, strain *E-clo-Niagara* plated on MacConkey agar; (**e**) Bacterial growth curves show that spermine (black circles) but not spermidine (black squares) inhibits the growth of *Enterobacter cloacae*, strain *E-clo-Niagara*. The IC_50_ for growth inhibition by spermine was 1.6 mg/mL. In contrast, a strain of *Serratia marcescens* was not inhibited at all by spermine (open circles).

The pattern of spermine production was strikingly different from all other detected metabolites. It was secreted exclusively by the host in a polarized way (apically) and its apical concentration was slightly lower in the presence of Nissle. Published data from the 1950s suggest that spermine has anti-bacterial properties and inhibits the growth of *E. coli* [52,53]. We hypothesize that if spermine has bactericidal effects, the spermine-enriched ApCM, but not spermine-free BlCM, will significantly inhibit bacterial growth. Indeed, incubation of Nissle 1917 (Figure 7a) and the most common human *E. coli* strain HS4 (Figure 7b) with ApCM significantly inhibited the growth of both bacteria, similarly to the commonly used cell culture standard penicillin + streptomycin combination to which the tested bacterial strains do not have a resistance. In contrast, basolateral media has no effect on bacterial growth.

To further prove that spermine is the apical constituent that affects bacterial growth, we used purified spermine to test its effect on the growth of laboratory strains and human pathogens. In solid agar assays, spermine substantially inhibited the growth of the *E. coli* laboratory strain JLM281 as well as human pathogenic bacteria *Enterobacter cloacae* (Figure 7c). Importantly, spermine-resistant inlay colonies were detected in both strains. In contrast, spermidine did not affect the bacterial growth. To quantify the polyamine effects on bacterial growth, liquid cultures of nosocomial human pathogens *Enterobacter cloacae*, strain *E-clo-Niagara* and *Serratia marcescens* were subjected to spermine or spermidine in a dose-dependent treatment (Figure 7d). Spermine, but not spermidine, was very effective in inhibiting the growth of *Enterobacter cloacae*. However, it did not affect the *Serratia marcescens* growth, indicating spermine specificity and bactericidal limitations.

### 2.7. Assessment of Epithelial Integrity of HCM Infected for More than 24 h with Low Initial Bacterial Concentration

**Figure 8:**
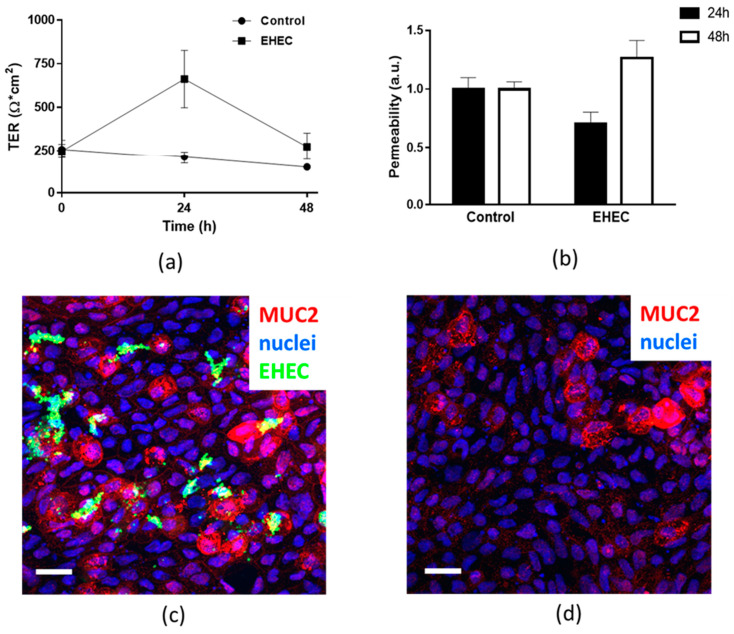
Infection of HCM with initial low inoculum of pathogenic EHEC does not destroy the epithelial layer at 48 h post-infection. Three days of differentiated HCM were apically infected with EHEC (10^3^ CFUs) for 48 h and the epithelial integrity was tested. (**a**) TER of EHEC-infected HCM was similar to this in uninfected passage-matched control HCM; *n* = 8 per each condition; (**b**) Transepithelial permeability of HCM to 3 kDa dextran was similar between uninfected and EHEC-infected cultures. Data presented as mean ± SEM; *n* ≤ 3 per each tested conditions; Representative maximum intensity projection images of differentiated HCM either (**c**) infected for 48 h with EHEC or (**d**) uninfected control; bar—20 µm.

Our metabolome data indicate that several metabolites are differently distributed between apical and basolateral compartments (e.g., Asp, succinic acid). This might be due to tight junctions (TJs) that create a tightly regulated diffusion barrier between colonic luminal and serosal content for ions, small and large molecules [54,55]. Dysfunctional TJ contributes to multiple diseases, such as ulcerative colitis [55]. It is well known that pathogenic bacteria and some commensals might contribute to the loss of TJ integrity leading to transepithelial movements of bacterial toxins, such as lipopolysaccharides. Interestingly, our metabolome data indicate that the apical-to-basolateral and vice versa gradients of some small molecules are also present in bacteria-infected HCM. Indeed, we infected HCM with a low initial bacterial concentration (10^3^ CFUs) for 48 h with an expectation that such conditions would allow testing of the host–bacterial cross-talk in a static system (in the absence of perfusion that would decrease the bacterial burden) without destroying the integrity of the epithelial layer. To test whether low initial bacterial inoculum allows the monitoring of host–microbial interactions over a prolonged infection time, we infected apically HCM with pathogenic EHEC (10^3^ CFUs) and monitored the epithelial integrity by measuring TER, permeability to high molecular weight dextran, and by examination of the monolayers via confocal microscopy. The TER of EHEC-infected HCM measured 24 and 48 h post-infection was not different from this in uninfected controls (Figure 8a). The apical-to-basolateral movement of 3 kDa AlexaFluor 680-labeled dextran was also similar between uninfected and infected monolayers (Figure 8b). These data suggest that in our experimental conditions, EHEC did not compromise the intestinal epithelial integrity for at least 48 h of infection. Examination of the randomly chosen fields of view from 48 h EHEC-infected HCM showed that bacteria were mainly concentrated near Mucin-2 (MUC2)-positive goblet cells (Figure 8c), as it has been previously shown [12,14]. Importantly, we found no gaps between the nuclei of the host cells in the EHEC-infected HCM, indicating no obvious massive cell death due to the infection. The overall morphology of EHEC-infected monolayers was similar to uninfected passage-matched cultures (Figure 8c,d), supporting our findings by TER and permeability measurements. We concluded that infection of static epithelial cultures with low initial bacterial inoculum might be a useful approach to monitor host–bacterial communication over a long time (days) without instant killing of epithelial cells by bacteria.

## 3. Discussion

In the present study, using a model of human colonic epithelium, we characterized the composition of small molecules released by colonoid monolayers into the apical and basolateral compartments. Using HCM co-cultured with apically added probiotic *E. coli* strain Nissle 1917, we further characterized the changes in small molecules due to host–bacterial interactions. Adult stem cell-derived HCM is one of the most advanced models of colonic epithelial tissue that preserves in a culture the whole composition of epithelial cells, including mucus-producing goblet cells, enteroendocrine, and other types of rare cells in a physiological ratio [14,15,56]. Depending on the composition of external growth factors, the HCM could be fully undifferentiated, representing mainly stem and transiently amplifying cells of the colonic crypts that include the precursors of secretory and absorptive cell lineages. HCM could also be fully differentiated and produce a thick apical mucus layer, as it occurs at the colonic surface, or represent a partial differentiation stage.

We found that apical exposure of differentiated mucus-producing HCM to Nissle 1917 changes not only apical but also the basolateral composition of the small molecules. This is not due to Nissle-induced epithelial damage that may lead to the leak of metabolites across the epithelium, because the basolateral concentration of several metabolites, including the mammalian toxins, 2-oxoisovaleric acid, and creatinine, are substantially reduced in the basolateral compartment in the presence of apical bacteria. Our data suggest that the infection of human epithelial cells with an initial low bacterial concentration (commensal or pathogenic, Figure 8) might be a useful approach to study host–bacterial co-existence and communication, including the detection of host-defense mechanisms and the bacterial approaches to evade the host responses. This is in contrast to intestinal epithelial infection with high bacterial concentrations (10^6^ and above) that often lead to epithelial disintegration and cell death [14]. These data further illustrate how the microbiome influences the physiology on local and systemic levels [1,2,3,4,5,6,7,8,9,10,11].

Several recent excellent reviews discussed in depth the role of intestinal flora and commensal-derived soluble factors in the development and progression of human diseases as well as their role as probiotics, prebiotics, and bacterial metabolites in human health, both intestinal and systemic [1,2,3,4,5,6,7,8,9,10,11]. The role of commensals in human health and diseases is supported by numerous associative investigations and correlative data spurred mainly by technological advances that combine sequencing, use of germ-free or antibiotic-treated animal models, or fecal material transfer therapies. Mechanistic studies based on identification and functional testing of microbe-secreted molecules improved our understanding of how the intestinal flora modulates host physiology and helped to develop the therapeutic strategies to mitigate disease. However, less is known about the human intestinal epithelium-secreted causal molecules that mediate these microbe–host interactions. MUC2 is probably one of the most studied epithelia-secreted proteins. MUC2 builds the colonic mucus layers occupied from the luminal side by commensal bacteria. Mucus serves as the physical barrier that protects the epithelium from harmful luminal contents and prevents the commensals from reaching the cell surface, which might lead to a harmful immune response [57,58]. Additionally, mucus serves as the inter-species communication ground and this exchange of information occurs mainly by microbe- and host-derived soluble molecules. While the protein composition of human colonic mucus in health and disease recently drew a lot of attention [58], the mucus metabolome is less characterized. Using human colonic mucus samples isolated from either biopsies or resected tissue, it is relatively simple to determine whether identified protein is derived from the host or microbes. Though it is not as simple in the case of small molecules, because the same metabolites can be generated either by the host or bacteria, or both, and some could be generated extracellularly by biochemical reactions between secreted enzymes and their substrates inside the mucus. Mouse germ-free models could be informative, but there are numerous differences between mouse and human gut physiology and pathologies, as well as differences in microbial communities and colonization patterns.

Our data suggest that mucus-producing HCM might be useful in the identification of host-derived metabolites. HCM-bacterial co-cultures could be informative in characterizing the effect of either a single strain or microbial community on the composition and relative amount of small molecules. It also might be useful to study the complex process of molecular exchange between the host and bacteria, and the extracellular enzymatic conversion of the metabolites.

Another novel finding is the existence of a broad antibacterial host defense mechanism implemented by apically secreted metabolites, specifically spermine. The antibacterial effects of polyamines, especially spermine, were recognized more than six decades ago [52,53]. Despite the promising start, research on polyamines in host–microbe interactions fell into a long lull from the 1980s to the 2000s. Recently, a mini-renaissance of interest in polyamines and microbial pathogenesis may have begun [59,60,61]. ApCM is a mixture of colonoid-secreted molecules and mucus. However, the classical anti-microbial peptides, including cathelicidin, and β-defensins 1 and 2, were not reported in the human colonic mucus proteome database [58]. Our data suggest that mucus, in addition to being a physical barrier for numerous luminal commensals lacking the enzymes necessary to degrade the dense attached mucus layer, also represents a chemical barrier filled with spermine and possibly other bactericidal small molecules, thus keeping the bacteria away from the cell surface. We found that although spermine shows broad bactericidal properties in vitro, there are spermine-resistant bacterial strains. Additionally, spermine-sensitive strains quickly generate spermine-insensitive clones in vitro. If confirmed in vivo, spermine and possible other anti-bacterial metabolites (host-secreted or diet-derived) might substantially influence the composition of colonic flora.

To summarize, human colonic epithelium communicates with the microbes via small molecules, including host-secreted anti-bacterial factors that might substantially shape the commensal and pathogenic colonic flora. Commensals affect the secretion of host metabolites and modulate the transepithelial delivery of small molecules, contributing to extra-epithelial intestinal and possibly systemic effects. HCM is a useful model to study host–commensal metabolic interactions. Our current studies were limited to two human colonoid lines. Thus, the commonality and difference in colonic epithelium-secreted metabolites due to host genetic and epigenetic factors as well as how the host genetic influences the colonic flora via small molecules have to be determined in the future research. Hypoxic conditions in human small intestinal and colonic lumen might also significantly modulate metabolite-dependent host–microbial communication. Although enrichment in hypoxia signaling in Transwell-grown duodenal and jejunal enteroid monolayers has been recently reported [62], the detailed contribution of gut segment-specific hypoxic conditions to the composition of small molecules in the lumen should be addressed in the future.

## 4. Materials and Methods

### 4.1. Reagents and Chemicals

Glutamine assay (Glutamine/Glutamate-Glo™; Cat# J8021) was purchased from Promega (Madison, WI, USA). SLC1A5 inhibitor GPNA (G6133), spermine, and spermidine were purchased from Sigma-Aldrich (St. Louis, MO, USA). Mouse monoclonal anti-MUC2 antibody (ab11197, Abcam; Cambridge, UK), Fluorescein-labeled BacTrace anti-EHEC antibody (KPL (LGC Seracare, Milford, MA, USA), secondary donkey anti-mouse AlexaFluor™ 568-labeled antibody, and 3 kDa Alexa Fluor™ 680-labeled dextran (Cat # 34681) were from Invitrogen (Waltham, MA, USA); Hoechst for DNA/nuclear staining (Thermo Fisher Scientific; Waltham, MA, USA).

### 4.2. Human Colonoid Monolayer Cultures

Colonic tissue samples from two different donors were obtained from discarded normal tissue of patients undergoing colorectal surgery. The protocol was approved by University of New Mexico IRB (protocol number 18-171) and the patients’ informed consents were obtained. Crypts were isolated from the de-identified tissue samples and colonoid cultures were established as we described before [12,13,14,63]. Briefly, cells were resuspended in Matrigel (Corning; Corning, NY, USA) and cultured in a complete medium (CM) with growth factors (CMGF^+^ expansion medium). CM media contained Advanced Dulbecco’s modified Eagle medium/Ham’s F-12 (Life Technologies; Carlsbad, CA, USA), 100 U penicillin/streptomycin (Life Technologies; Carlsbad, CA, USA), 10 mM HEPES (Life Technologies; Carlsbad, CA, USA), and 0.2 mM GlutaMAX (Life Technologies; Carlsbad, CA, USA). CMFG+ medium is CM medium supplemented with 50% *v*/*v* Wnt3A-conditioned medium, 15% *v*/*v* R-spondin-1-conditioned medium, 10% *v*/*v* Noggin-conditioned medium, 50 ng/mL human epidermal growth factor (EGF) (Life Technologies Carlsbad, CA, USA), 10 nM gastrin I (Sigma), 500 nM A-83-01 (Tocris Bioscience; Minneapolis, MN, USA), 10 μM SB202190 (Sigma-Aldrich; St. Louis, MO, USA), 1 × B27 supplement (Life Technologies; Carlsbad, CA, USA), 1 mM *N*-acetylcysteine (Sigma-Aldrich; St. Louis, MO, USA),10 µM CHIR99021 (Tocris Bioscience; Minneapolis, MN, USA), and 10 µM Y-27632 (Tocris Bioscience; Minneapolis, MN, USA), and Noggin (Cat # 120-10C; PeproTech, Cranbury, NJ, USA). CHIR99021 and Y-27632 were removed from CMGF^+^ media during subsequent media replacements. Conditioned media was obtained from the following cell lines expressing the growth factors: Wnt3A (ATCC CRL-2647 cells (American Tissue Culture Collection), R-spondin-1 (kindly provided by Dr. Calvin Kuo, Stanford University, Palo Alto, CA, USA).

Two colonoid cultures were passaged every 10–14 days for expansion or monolayer cultures. Colonoid monolayers were prepared as we previously described in detail [12,13,14,63]. Briefly, colonoid fragments resuspended in CMGF+ media were plated on Transwell filters (0.4 µm pore, polyester membrane, 0.33 cm^2^ surface area; Cat # 3470, Corning, Glendale, AZ, USA) coated with 10 µg/cm^2^ human collagen IV solution (C5533, Sigma-Aldrich, St. Louis, MO, USA). Transepithelial electrical resistance (TER) was measured using an EVOM2 voltohmmeter (World Precision Instruments) to monitor confluency of the monolayers.

To differentiate the Transwell-grown confluent human colonoid monolayers (HCMs), the expansion medium was exchanged with the differentiation (Diff) medium (Advanced DMEM/F12, Cat # 12-491-015, Fisher Scientific, Waltham, MA, USA; GlutaMax, Cat # 35-050-061, Fisher Scientific, Waltham, MA, USA; HEPES, Cat # MT25060CI, Fisher Scientific, Waltham, MA, USA) supplemented with B27 (Cat # 17-504-044, Fisher Scientific, Waltham, MA, USA), N-Acetyl-L-cysteine (Cat # A9165, Sigma-Aldrich, St. Louis, MO, USA), human EGF (Cat # 236-EG-01, R&D Systems, Minneapolis, MN, USA), [Leu15]gastrin (Cat # G9145, Sigma-Aldrich, St. Louis, MO, USA), A83-01 (Cat # 2939, Tocris BioScience, Minneapolis, MN, USA), and SB202190 (Cat # S7067, Sigma-Aldrich, St. Louis, MO, USA). The differentiation medium was changed every 2-3 days and the differentiation continued for 5 days, as we have described [12,13,14,63].

### 4.3. Bacterial Strains and the Apical Infection of HCM

*E. coli* HS4 and probiotic Nissle 1917 were obtained from Dr. Edgar Boedeker, U. New Mexico. We used genetically well-characterized EHEC O26:H11 strain 97-3250 obtained from Dr. S. R. Leonard, FDA as a representative human EHEC pathogen [12,19]. The aliquots of each bacterial strain from overnight culture were diluted in either Diff media, or in ApCM, or in BlCM (total volume 100µL) to reach a final concentration of 10^3^ CFU; the bacteria-containing aliquots were either applied to the apical surface of HCM or incubated without cells. Culture turbidity, as OD_600_, was measured.

E-clo-Niagara strain from *Enterobacter cloacae* species [64], *Serratia marcescens*, and *E. coli* laboratory strain JLM281 [65] were from Dr. John K. Crane laboratory. Culture turbidity, as OD600, was measured.

In order to apically infect the differentiated HCM, a sterile inoculation loop was used to pick up a small amount of bacteria (Nissle of EHEC) from a glycerol stock and transfer to 2 mL of LB broth (Cat # BP9723-500, Fisher Scientific, Waltham, MA, USA). The bacterial culture was grown overnight at 37 °C on an orbital shaker (Shel-Lab 1570, Cornelius, OR, USA). In total, 50 μL of the original starter culture were resuspended in 5 mL of fresh LB broth and incubated for 3–5 h to obtain a log phase culture (OD_600_ = 0.4–0.6). Then, the culture was spun down at 12,000 for 10 min using Heraeus Fresco 17 Centrifuge from Fisher Scientific (Waltham, MA, USA). Supernatant was removed and bacteria were resuspended in differentiation media. Bacterial suspension was added to the apical comportment of the HCM that contained 100 μL of the differentiation medium (final concentration 10^3^ CFUs). The HCM were incubated at 37 °C in a tissue culture incubator (Cat # 10810-744, VWR, Aurora, CO, USA).

To measure the OD_600_, 20 μL of the original culture were put into 980 μL of differentiation media and mixed in a small tube, then transferred into the disposable cuvettes made of clear plastic with a light transmission of 300 to 900 nm (Fisher Scientific Catalog No.14-281-215) and OD was measured using a SmartSpec 3000 Spectrophotometer (BioRad, Hercules, CA, USA). CFU was calculated based on the formula OD_600_ of 1.0 = 8 × 10^8^ cells/mL and multiplied by the dilution factor of 50.

### 4.4. Media Sample Preparation for Metabolomic Analysis

Apical (100 μL) and basolateral (600 μL) media collected from the last two days of differentiation from uninfected HCM (termed apical and basolateral conditioned media) were subjected to metabolome analysis. Similarly, apical and basolateral conditioned media from the bacteria-infected HCM were collected for metabolome analysis.

Two independent samples of ApCM and BlCM collected from either uninfected or Nissle-infected for 48 h HCM, as well as sample of Nissle infected for 48 h Diff media, and uninfected Diff media were subjected to metabolome analysis. The 100 µL samples were sent from University of New Mexico to Human Metabolome Technologies, Inc. (Boston, MA, USA). Metabolome analysis was performed using Capillary Electrophoresis Time-of-Flight Mass Spectrometry (CE-TOFMS) in two modes for cationic and anionic metabolites. At HMT, 40 μL of sample were mixed with 10 μL of Milli-Q water containing internal standards (1000 μM). The mixture was then filtrated through a 5-kDa cut-off filter (ULTRAFREE-MC-PLHCC, HMT, Yamagata, Japan) to remove macromolecules. In total, 190 metabolites were detected (144 metabolites in cation mode and 46 metabolites in anion mode) on the basis of HMT, Inc. standard library. Here, we analyzed the metabolites that were present in µM concentrations.

### 4.5. Glutamine ELISA Assay

Three days of differentiated HCM were divided into control and experimental groups. The control HCM were incubated for 2 days in differentiation media plus GPNA solvent (DI water), while experimental HCM were treated with SLC1A5 inhibitor (100 μM GPNA; Cat # G6133; Sigma-Aldrich; St. Louis, MO, USA). Apical or basolateral media from HCM were collected and glutamine was measured using a bioluminescent assay according to the manufacturer’s protocol. Briefly, 25 μL aliquots of apical and basolateral media were transferred into a white 96-well luminometer plate. Two sets of wells were prepared to determine luminescence: (1) total glutamine and glutamate and (2) glutamate only. In total, 25 μL of glutaminase enzyme solution were added to the first set of wells and 25 μL of glutaminase buffer only with no glutaminase were added to the second set of wells. The plate was shaken on an Orbit^TM^ 300 Digital Multipurpose shaker (Cat # S2030-300, Labnet, Edison, NJ, USA) for 1 min followed by incubation at room temperature for 40 min. Then, 50 μL of glutamate detection reagent were added to all wells. The plate was shaken for 1 min using an Orbit^TM^ 300 Digital Multipurpose shaker and incubated at room temperature for 60 min. Luminescence was recorded using a luminometer the Synergy/Neo2 multi-mode plate reader from BioTek (Winooski, VT, USA). Glutamine levels were calculated by subtracting glutamate only recordings from the total glutamine and glutamate recordings.

### 4.6. Polyamine Effects on Bacterial Growth on Solid Agar

Strains were diluted 1:100 into sterile normal saline from overnight culture, then streaked onto MacConkey agar plates using a sterile swab and a criss-cross pattern. In total, 10 µL of a 50 mg/mL solution of spermine or spermidine were spotted onto sterile blank 6 mm paper disks (BD, VWR 90002-114) and the plates were incubated at 37 °C for 16 h, then photographed using a Macro lens.

### 4.7. Quantitative Measurements of the Effect of Polyamines on Bacterial Growth

First, 500 mg/mL of spermine or spermidine stock solution was prepared by dissolving 500 mg of each polyamine (Cat # 85590 and # S2626, respectively, Sigma-Aldrich, St. Louis, MO, USA) in DI water (Cat # W4502, Sigma-Aldrich, St. Louis, MO, USA).

Strains were subcultured at a dilution of 1:100 from an overnight culture into antibiotic-free DMEM medium (Gibco Division of Thermo-Fisher; Waltham, MA, USA). Two-fold serial dilutions of the polyamines spermine or spermidine were made in 96 well plates, and growth continued at 37 °C with 500 rpm shaking in a Bioer MB-101 shaker-incubator (Pro Lab Supply Corp, Hialeah, FL, USA). Culture turbidity, as OD_600_, was measured at 4 h of growth.

### 4.8. Immunofluorescence Staining and Microscopy

HCM apically infected for 48 h with EHEC (10^3^ CFUs) or passage-matched uninfected controls were processed for immunofluorescence after ApCM and BlCM collection. HCM were washed 3 times with cold PBS and fixed with 4% paraformaldehyde (Electron Microscopy Sciences, Hatfield, PA, USA). Fixed cultures were permeabilized with the mixture of permeabilized and blocked for 1h with PBS containing 15% fetal bovine serum, 2% BSA, and 0.1% saponin, all at room temperature (RT). Primary antibody (Ab) against MUC2 (1:100 in PBS) was applied overnight at 4 °C and the monolayers were washed 4 × 15 min in cold PBS. Next, the cells were incubated in the mixture of secondary fluorescent antibody (1:100 in PBS), EHEC-specific fluorescent antibody (1:100 in PBS), and Hoechst (1:1000 in PBS) at RT for 1 h, and then washed 4 × 15 min in cold PBS. Filters were cut out and mounted in FluorSave reagent (Millipore Sigma; Burlington, MA, USA) on glass slides for examination by microscopy.

The 0.5 µm confocal optical sections of the EHEC-infected and control HCM (*n* = 3 HCM per each condition) were collected using Zeiss 800 LSM (Zeiss, Oberkochen, Germany) available through the Fluorescence Imaging Core of the U. New Mexico Comprehensive Cancer Center. The maximum intensity projections from the acquired images were generated using Zen 2012 edition image processing software (Zeiss).

### 4.9. Epithelial Permeability Assay

Transepithelial movement of AlexaFluor-680-dextran was measured to determine whether EHEC infection for 48 h caused the disruption of colonoid tight junctions leading to the increase in monolayer permeability. Dextran (1:20 in Diff. media) was added to the apical side of HCM (EHEC-infected or uninfected control). Basolateral media (100 µL) was sampled at 24 and 48 h, and the sampled volume was replenished with fresh Dif. media. The dextran content in the basolateral samples was measured by relative fluorescence intensity using the Synergy/Neo2 multi-mode plate reader from BioTek (Winooski, VT, USA).

### 4.10. Statistical Analysis

Statistical analyses were performed using either Excel (Microsoft) or Prism (Graphpad). Unpaired Student’s *t*-test was used to analyze statistical differences, with *p* < 0.05 considered statistically significant.

## Data Availability

Data available within article and Supplementary Materials.

## Supplementary Materials

The following are available online at https://www.mdpi.com/article/10.3390/metabo11120841/s1, Figure S1: In contrast to differentiated HCM, undifferentiated colonoids significantly decreased the relative Gln concentration in undifferentiation (growth) media, Table S1: List of metabolites detected in µM concentrations in media collected from six experimental conditions, Table S2: Nissle produced metabolites that were not detected in other tested conditions.
