# Supplementary material for: Cross-Talk between Probiotic Nissle 1917 and Human Colonic Epithelium Affects the Metabolite Composition and Demonstrates Host Antibacterial Effect
Source: Metabolites. 2021 Dec 5;11(12):841. doi: 10.3390/metabo11120841 (PMC8706777; doi:10.3390/metabo11120841)
Supplement: Supplementary file 1 [file metabolites-11-00841-s001.zip › metabolites-1443956-supplementary.pdf]

Supplemental Data

Table S1.

List of metabolites detected in µM concentrations in media collected from six experimental conditions

	Experimental Conditions									
	Diff media Control (n=1)	Nissle in DF media (n=1)	ApCM (AVG; n=2)	SD	BlCM (AVG; n=2)	SD	**APCM + Nissle (AVG; n=2)**	SD	BlCM + Nissle (AVG; n=2)	SD
	Metabolites, in µM									
**Amino Acids**										
Glutamine	3.8	1.1	649.5	169.0	712.0	58.0	1.9	2.6	278.0	392.0
Alanine	52.0	59.0	1459.5	89.8	1037.0	120.2	214.0	295.6	687.1	967.3
Glutamate	47.0	31.0	23.5	0.7	52.5	0.7	67.0	17.0	269.0	359.2
Proline	140.0	1.4	539.5	102.5	401.0	84.9	92.1	127.1	146.5	205.8
Glycine	257.0	6.0	506.0	82.0	314.0	15.6	47.2	57.8	125.0	176.8
Phenylalanine	208.0	68.0	272.0	2.8	214.0	2.8	56.0	28.3	103.5	96.9
Aspartate	43.0	3.9	1.3	0.3	31.0	1.4	9.7	7.6	2.8	2.0
Histidine	146.0	66.0	212.5	10.6	150.0	1.4	64.0	21.2	83.5	55.9
Isoleucine	323.0	154.0	296.5	21.9	333.5	3.5	136.5	19.1	203.0	100.4
Serine	134.0	2.8	159.0	32.5	108.5	10.6	3.0	0.1	–	–
Valine	361.0	137.0	453.0	1.4	378.5	10.6	150.0	63.6	214.0	145.7
Leucine	420.0	82.0	359.5	44.6	413.0	9.9	83.5	43.1	191.0	207.9
Lysine	630.0	9.9	992.0	77.8	626.0	1.4	79.1	108.8	245.8	346.8
Methionine	76.0	0.6	52.0	4.2	69.5	2.1	0.9	0.4	19.1	26.8
Tryptophan	11.0	0.2	14.0	2.8	15.0	0.0	0.3	0.1	5.5	7.8
Tyrosine	183.0	76.0	259.0	4.2	189.0	1.4	57.5	19.1	94.5	79.9
Asparagine	38.0	0.0	47.5	3.5	34.5	2.1	–	–	–	–
Threonine	429.0	0.0	618.0	14.1	442.0	4.2	1.3	0.2	110.0	155.6
Arginine	879.0	402.0	1303.5	55.9	854.0	7.1	405.5	88.4	538.5	236.9
β-Alanine	–	1.7	0.9	1.3	–	–	–	–	–	–
Homoserine	–	–	–	–	–	–	1.0	0.2	0.7	0.9
**Nucleosides and Nucleoside Precursors**										
Uracil	–	6.0	2.5	3.5	–	–	10.7	1.8	–	–
Guanosine	–	0.8	0.1	0.1	–	–	0.4	0.1	0.3	0.2
Uridine	3.1	2.1	8.0	2.2	4.2	0.4	2.9	0.1	2.6	0.8
Inosine	–	1.3	0.9	0.4	–	–	5.2	0.9	0.5	0.6
Ribulose 5-phosphate	–	–	–	–	–	–	3.2	4.5	–	–
**Organic Acids**										
3-Hydroxybutyric acid	–	–	77.0	26.9	21.5	4.9	39.0	22.6	19.0	26.9
Citric acid	–	25.0	147.0	82.0	52.5	9.2	30.0	7.1	56.0	12.7
Succinic acid	–	33.0	15.0	21.2	–	–	69.5	98.3	317.0	448.3
Lactic acid	55.0	6920.0	25185.5	3155.8	17728.5	3247.7	25.5	0.7	8158.0	11508.9
GABA	–	234.0	79.0	39.6	23.0	9.9	205.0	168.3	192.0	230.5
**Toxins**										
2-Oxoisovaleric acid	–	–	12.6	4.9	4.1	5.7	6.5	9.2	–	–
Creatinine	–	–	0.4	0.1	0.2	0.2	0.2	0.3	–	–
**Polyamines**										
Spermine	–	–	7.5	2.1	–	–	3.0	1.4	–	–
Spermidine	–	13.0	0.9	0.2	0.4	0.1	14.0	2.8	3.3	2.2
Putrescine	94.0	270.0	–	–	14.5	20.5	201.0	32.5	109.0	79.2

Table S2. Nissl produced metabolites that were not detected in other tested conditions.

Metabolites	μM
3-Phosphoglyceric acid	27
Acetyl CoA_divalent	2.1
AMP (adenosine monophosphate)	4.2
UMP (Uridine monophosphate)	2.8
Glycerol 3-phosphate	6.3
CMP (cytidine monophosphate)	4.7
NAD+	4.5

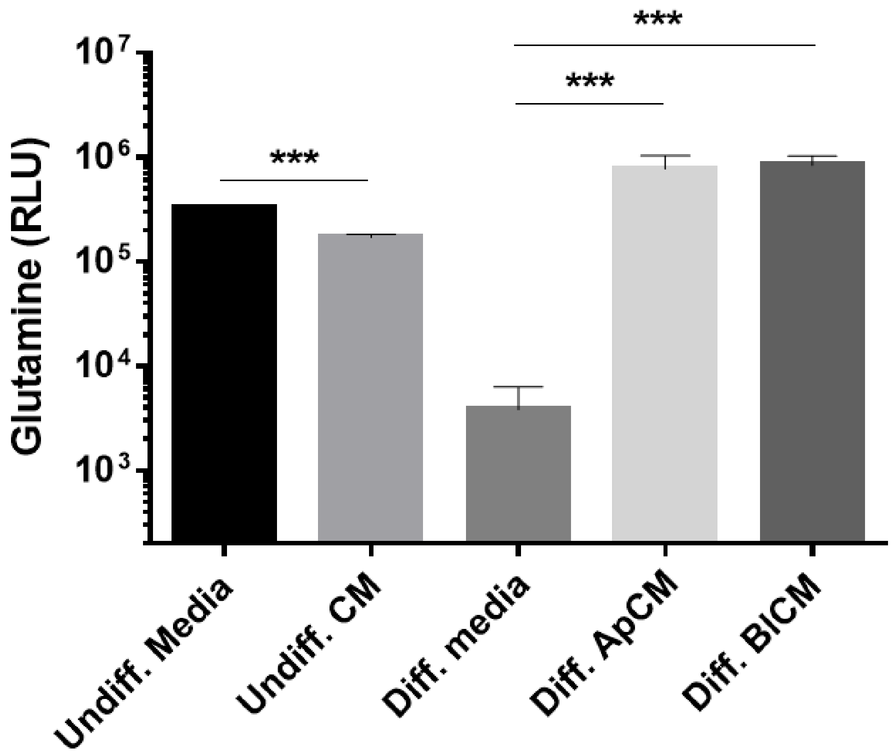

Figure S1. In contrast to differentiated HCM, undifferentiated colonoids significantly decreased the relative Gln concentration in undifferentiation (growth) media. Glutamine relative luminescence was measured in growth (Undiff.) media, Undiff. media conditioned by colonoids (Undiff. CM), Diff. media, and ApCM and BlCM from differentiated colonoids. Data presented as mean ±SEM, n=3 per each tested condition; *** p≤0.01
